# A Randomized Controlled Trial on Functional Relaxation as an Adjunct to Psychoeducation for Stress

**DOI:** 10.3389/fpsyg.2017.01553

**Published:** 2017-09-27

**Authors:** Claas Lahmann, Maria Gebhardt, Heribert Sattel, Andreas Dinkel, Christoph Pieh, Thomas Probst

**Affiliations:** ^1^Department of Psychosomatic Medicine and Psychotherapy, University Medical Center Freiburg, Faculty of Medicine, University of Freiburg, Freiburg, Germany; ^2^Department of Psychosomatic Medicine and Psychotherapy, Klinikum Rechts der Isar, Technical University of Munich, Munich, Germany; ^3^Department for Psychotherapy and Biopsychosocial Health, Danube University Krems, Krems, Austria; ^4^Department of Psychosomatic Medicine, University Hospital Regensburg, Regensburg, Germany; ^5^Georg-Elias-Müller Institute for Psychology, Georg-August University of Göttingen, Göttingen, Germany

**Keywords:** stress, stress reduction, functional relaxation, psychoeducation, randomized controlled trial

## Abstract

This randomized controlled trial investigated whether adding the psychodynamically based body-oriented psychotherapy “Functional Relaxation” (FR) to psychoeducation (PE) is more effective than PE alone to reduce stress and stress-associated complaints. Eighty-one participants with elevated stress-levels, ≥50 points on the global scale of the Perceived Stress Questionnaire (PSQ), received either 10 sessions of manualized FR + PE (*n* = 42) or two sessions of manualized PE alone (*n* = 39) in a group setting. Six FR trainers took part in this study. Stress-level (PSQ) was the primary outcome and secondary outcomes were depression (PHQ-9) and somatization (PHQ-15). Multilevel models for discontinuous change revealed that FR + PE was more helpful to reduce stress-levels than PE from pre-treatment to post-treatment (t0 → t1) as well as from pre-treatment to 6-month follow-up (t0 → t2) (both *p* < 0.05) with effect sizes (*d*) being medium for PE (*d*_t0 → t1_ = 0.57; *d*_t0 → t2_ = 0.67) and large for FR + PE (*d*_t0 → t1_ = 1.57; *d*_t0 → t2_ = 1.39). Moreover, FR + PE affected depression and somatization more positively than did PE from t0 to t1 as well as from t0 to t2 (all *p* < 0.05). Effect sizes for depression were small to medium for PE (*d*_t0 → t1_ = 0.52; *d*_t0 → t2_ = 0.37) and large for FR + PE (*d*_t0 → t1_ = 1.04; *d*_t0 → t2_ = 0.95). Effect sizes for somatization were small for PE (*d*_t0 → t1_ = 0.18; *d*_t0 → t2_ = 0.19) and medium to large for FR + PE (*d*_t0 → t1_ = 0.73; *d*_t0 → t2_ = 0.93). In summary, the combination of FR and PE was more effective than PE alone. The results of the present trial provide first evidence of FR as a potent component of stress interventions. Adding FR to such interventions might better help prevent clinically relevant disorders such as depression or somatization.

## Introduction

The Stress in America™ survey 2015 (American Psychological Association, 2016) revealed that 24% of the adult population experienced high stress-levels and that 34% perceived increased stress during the last year (vs. 16% who reported decreased stress). These are alarming results given the fact that a high stress-level is a risk factor for several metabolic and mental disorders (e.g., Cohen et al., 2007; Lupien et al., 2009; Provencal and Binder, 2015), more specifically, for example, for cardiovascular disease (e.g., Dimsdale, 2008; Steptoe and Kivimäki, 2012; Booth et al., 2015) and depression (e.g., van Praag, 2004; Wang, 2005; Vinkers et al., 2014).

These results underline the relevance of stress management/coping. Folkman and Lazarus (1980, 1985) distinguished problem-focused (addressing the source of the stress) from emotion-focused (tackling the stress-induced emotions) coping. Another distinction is dysfunctional vs. functional coping. A dysfunctional coping strategy leads to problems even though the stress-level may be reduced, whereas a functional coping strategy has no harmful effects. The functionality of certain coping strategies also depends on how extensively they are applied: They might be functional in the short-term, but dysfunctional in the long-term (e.g., repression).

Behavioral and mental disengagement (Carver et al., 1989) or self-harm (e.g., Chapman et al., 2006; Kleindienst et al., 2008) and related drug abuse (e.g., Brady and Sonne, 1999; Sinha, 2001) as well as unhealthy eating behavior (e.g., Greeno and Wing, 1994; Torres and Nowson, 2007) are examples of dysfunctional coping. Functional coping includes, for example, exercising mindfulness (e.g., Chiesa and Serretti, 2009; Khoury et al., 2015), practicing relaxation techniques (e.g., Carlson and Hoyle, 1993; Ernst and Kanji, 2000; Chiesa and Serretti, 2009), taking part in psychoeducation (PE) (e.g., Donker et al., 2009; Van Daele et al., 2012), or being physically active (e.g., Penedo and Dahn, 2005; Warburton et al., 2006).

Many of these functional coping strategies make use of bodily processes. For example, tensing and relaxing the muscles of different body parts is essential in progressive muscle relaxation, and several mindfulness-based interventions (e.g., body-scan, breathing meditation, yoga) rely on observing and appreciating body sensations (see Burg et al., 2017). Another intervention with a body focus is “Functional Relaxation” (FR; Fuchs, 2013). FR originally belongs to the psychodynamically based body-oriented psychotherapy methods most frequently used in Germany, Austria, and Switzerland. Minute movements of small joints, hardly noticeable to observers, are performed during relaxed expiration, accompanied by observing differences of body feelings triggered by these movements. Thereby, FR focuses on the discovery of proprioception by exploring the perceived differences of body sensations and the finding of one's own rhythm, aiming at rebalancing the autonomous nervous system triggered by small movements of the joints. In contrast to exercise-based methods such as, progressive muscle relaxation, and in accordance with the theoretical foundation in psychodynamic psychotherapy, the resulting sensations are described either nonverbally or explicitly during the therapeutic interaction.

Up to now, studies on FR solely rely on clinical samples and the results support the efficacy of FR for the following disorders: Somatoform heart disorder (Lahmann et al., 2008a), asthma (Loew et al., 1996, 2001; Lahmann et al., 2009, 2010a), irritable bowel syndrome (Lahmann et al., 2010b), chronic headache (Loew et al., 2000), and dental anxiety (Lahmann et al., 2008b).

Although these studies have shown that FR can reduce clinically relevant problems, it remains unclear whether FR is an effective intervention for stress in non-clinical samples. Reducing stress in non-clinical samples is important as this might prevent clinically relevant problems. Therefore, this randomized controlled trial was conducted, which compared the combination of FR and psychoeducation (PE) with PE alone in individuals with elevated stress-levels. The hypotheses were that the condition offering FR + PE is superior to a condition with PE alone at the end of the intervention as well as at 6-month follow-up to reduce stress-levels (primary outcome) as well as depression and somatization (secondary outcomes).

## Materials and methods

The Ethics Committee of the Technical University of Munich approved the trial and the applied materials and methods. The study was conducted in accordance with the approved guidelines. Written informed consent was obtained from all subjects in accordance with the Declaration of Helsinki.

The materials and the data are available upon request from the first author.

### Study design

The present study is a randomized controlled trial with one between-subject factor (condition with two levels: FR + PE vs. PE) and one within-subject factor (measurements point with three levels: pre-treatment, post-treatment, 6-month follow-up).

A researcher not otherwise involved in the study generated a blocked randomization list by a computer program and applied this list to the sample. After receiving informed consent from a participant, a randomization request was sent from the study therapist to the independent researcher and the result for the participant in question was returned to the therapist within 24 h. According to this result, the participant was allocated to the FR + PE or the PE alone condition.

The sample size calculation was done for small to moderate effect sizes; the initial plan was to randomize *N* = 128 participants to the two conditions (*n* = 64 to FR + PE; *n* = 64 to PE alone). Each therapist was intended to treat *n* = 16 participants (*n* = 8 with FR + PE; *n* = 8 with PE alone). Due to the explorative character of the study (first one to apply FR for stress), no concrete power calculation was performed.

### Participants

Participants were recruited by flyers in family practices or by the participating therapists. Of all persons interested in participating in the study, only those scoring ≥50 points on the global scale of the “Perceived Stress Questionnaire” (PSQ) were included. The cut-off “≥50 points on the global scale of the PSQ” was used to define elevated stress-levels, since values equal or above 50 represent PSQ scores being at least one standard deviation (SD = 17) higher than the mean (*M* = 33) of the German population (Fliege et al., 2005). Exclusion criteria were age under 18, severe mental or physical disorders, and insufficient knowledge of the German language. One hundred and forty-five persons had interest to take part in the study, *n* = 95 participants could to be randomized to one of the two conditions, and *n* = 81 participants (*n* = 42 participants of the FR + PE condition, *n* = 39 participants of the PE alone condition) starting treatment were statistically analyzed.

**Figure 1:**
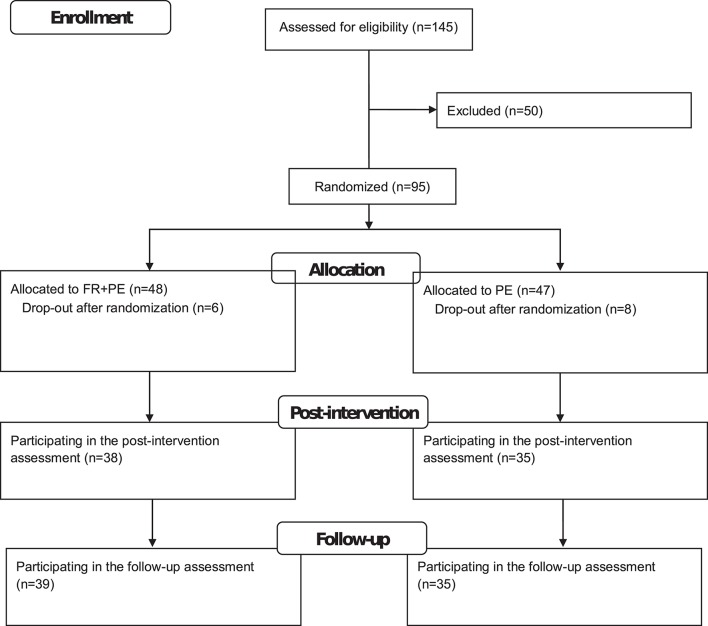
Flow diagram of the study.

The flow diagram is displayed in Figure 1. Of the *n* = 81 analyzed participants, *n* = 63 (77.8%) were female. A chi-squared test showed that the gender ratio was not significantly different between the two conditions [$\chi_{\left(1\right)}^{2}$ = 0.51; *p* = 0.48] with 81.0% female participants in FR + PE and 74.4% female participants in PE. The participants were on average *M* = 47.15 (SD = 11.39) years old. The two conditions did not significantly differ in their age [FR + PE: *M* = 45.93 and SD = 10.56; PE: *M* = 48.46 and SD = 12.22; *t*_(79)_ = 1.00; *p* = 0.32].

### Therapists

Six therapists trained in FR and certified by the Working Group for Functional Relaxation (“Arbeitsgemeinschaft für Funktionelle Entspannung”) took part in the study. Each therapist provided both conditions FR and PE. Of the *N* = 81 participants analyzed in this study, *n* = 15 were treated by therapist 1 (FR + PE: *n* = 8; PE: *n* = 7), *n* = 12 by therapist 2 (FR + PE: *n* = 6; PE: *n* = 6), *n* = 16 by therapist 3 (FR + PE: *n* = 8; PE: *n* = 8), *n* = 8 by therapist 4 (FR + PE: *n* = 4; PE: *n* = 4), *n* = 16 by therapist 5 (FR + PE: *n* = 9; PE: *n* = 7), and *n* = 14 by therapist 6 (FR + PE: *n* = 7; PE: *n* = 7).

### Interventions

Both interventions were provided in a group setting.

FR in combination with psychoeducation (PE): The FR + PE intervention of the current study comprised 10 weekly sessions (90 min each). Each therapist was instructed to provide the interventions according to a manual developed in previous studies (Loew et al., 1996, 2000, 2001; Lahmann et al., 2008a,b, 2009, 2010a,b) and adapted to stress-reduction. The therapists were supervised by a therapist experienced in FR after the fourth and the eighth session. The content of the sessions of the FR + PE condition is summarized in Table 1.

**Table 1:** Content of functional relaxation combined with psychoeducation.

**Session**	**Content**
1	Introduction round
	Discussion on expectations
	Contact to foundation
2	Contact to foundation
	Tensing and relaxing muscles
	Short-lecture on basics of stress
3	Contact to foundation
	FR principles: Rhythm and connection between breathing and moving
	Short-lecture on stress-reactions
4	Contact to foundation
	Imaginary journey
	Focus on shoulders and pelvis
	Applying principles of FR
	Short-lecture on stress management
5	Contact to foundation
	Applying principles of FR
	Focus on heart: Movement of warmth, energy flow, and heart beat
	Short-lecture on circadian rhythms
6	Contact to foundation
	Applying principles of FR
	Focus on neck, head, underjaw, mouth, teeth, shoulders: Tensing and relaxing, saying yes vs. saying no, encumbrances
	Short-lecture on tension and demands in daily life
7	Contact to foundation
	Applying principles of FR
	Focus on sitting bones, lower back, spine as inner support
	Short-lecture on support in daily life
8	Contact to foundation
	Applying principles of FR
	Focus on the three parts of the back
	Short-lecture on formulation of goals
9	Contact to foundation
	Applying principles of FR
	Different postures when sitting on a chair
	Short lecture on stress prevention
10	Contact to foundation
	Applying principles of FR
	Questions and wishes of the participants
	Discussion on expectations

Psychoeducation (PE): PE functioned as the control condition as it has been done in previous trials (e.g., Miklowitz et al., 2007). As PE has been shown to be effective to reduce stress (Donker et al., 2009; Van Daele et al., 2012), PE can be considered an active control. In the current study, PE comprised 2 sessions (90 min each), which were conducted in a time interval of approximately 6 weeks. PE relied on an unpublished manual developed by a FR therapist. The content of PE included lectures of the therapists on stress (first session: definition, consequences, prevalence; second session: prerequisites and examples of successful stress management) and discussions between the participants.

### Measures

The following questionnaires were administered to the participants at pre-treatment (t0), end of intervention (post-treatment: t1), and 6-months after t0 (follow-up: t2). Importantly, the time interval between t0 and t1 differed between the conditions (PE: approx. 6 weeks; FR + PE: 10–12 weeks) whereas the time interval between t0 and t2 was the same for both conditions (6-months).

Perceived Stress Questionnaire (PSQ; Levenstein et al., 1993; German version: Fliege et al., 2001, 2005): The global scale of the 20-item version of the PSQ was used to assess the stress-level as primary outcome. This 20-item version is a revised version of the original PSQ. The PSQ has been demonstrated to be reliable and valid (Fliege et al., 2001, 2005; Kocalevent et al., 2007). The Cronbach's Alpha coefficient at t0 was good in our sample, α = 0.84. Besides t0, t1, and t2, the PSQ was also applied at another assessment point before t0 to evaluate the inclusion criterion “PSQ global score of at least 50 points.”

Patient Health Questionnaire (PHQ; Spitzer et al., 1999; German version: Gräfe et al., 2004): The PHQ-9 (Kroenke et al., 2001) was used to operationalize depression and the PHQ-15 (Kroenke et al., 2002) to assess somatization as secondary outcomes. The PHQ is a reliable and valid questionnaire (Kroenke et al., 2010). In the sample of the current study, the Cronbach's Alpha values at t0 reached acceptable values of α = 0.72 (PHQ-9) and α = 0.78 (PHQ-15).

### Statistics

All statistical analyses were performed with SPSS 22. The inferential statistical tests were conducted two-tailed and the significance value was set to *p* < 0.05.

Multilevel models for discontinuous change (Singer and Willett, 2003; Göllner et al., 2010) were performed to evaluate whether the primary and secondary outcomes show a different change pattern between the two conditions either from pre-treatment (t0) to post-treatment (t1) or from pre-treatment (t0) to 6-month follow-up (t2). As described by Göllner et al. (2010), the three assessment points (t0, t1, t2) were coded into two contrast variables, with the pre-treatment measurement (t0) point being the reference. The intervention condition (FR + PE vs. PE) was added to the multilevel models as a dichotomous factor, whereby the PE condition functioned as the reference. Full maximum likelihood estimation was used and an unstructured variance-covariance matrix was applied. The multilevel models had two levels: Assessments as level-1 and participants as level-2. Therapists were not included as another level in the multilevel models since the variance component associated with therapists was only 0.8% and statistically insignificant (*p* = 0.78) as a null model revealed (estimate for therapists = 2.44 (SE = 8.62); estimate for participants = 84.31 (SE = 28.69); residual = 211.16 (SE = 25.64)).

Moreover, effect sizes (*d*) were calculated with the means (*M*) and standard deviations (SD) to quantify how much progress the conditions made on the primary and secondary outcomes from t0 to t1 as well as from t0 to t2. The formulas for the calculation of the effect sizes were (M_t0_–M_t1_)/SD_t0_ and (M_t0_–M_t2_)/SD_t0_. Values between 0.20 and 0.50 will be interpreted as small, values between 0.50 and 0.80 as medium, and values of at least 0.80 as large effects.

## Results

### Results for stress

Table 2 shows the results of the multilevel for discontinuous change, the effect sizes, and the means as well as the standard deviations for the primary outcome stress-level (PSQ). The insignificant “intercept^*^intervention” term indicates that the two conditions did not have significantly different stress-levels at t0 (64.17 vs. 59.49; *p* = 0.09). The negative and significant “time1” term (*p* < 0.05) shows that stress-levels were significantly reduced by PE from t0 to t1. The “time1^*^intervention” term reached statistical significance (*p* < 0.05) and the corresponding parameter estimate was negative. This means that FR + PE reduced stress-levels significantly more than PE from t0 to t1. While participants in PE reduced their stress-level on average 6.17 points on the PSQ at post-treatment, participants in FR + PE reduced their stress-level on average 20.98 (6.17 + 14.81) points on the PSQ.

**Table 2:** Results for the primary outcome stress-level measured with the global scale of the “Perceived Stress Questionnaire” (PSQ).

	**Parameter**	**Estimate (SE)**	**Statistics**
Results of the multilevel model	Intercept	59.49 (2.00)	*t*_(76.20)_ = 29.68; *p* < 0.01
	Intercept^*^ intervention	4.68 (2.76)	*t*_(76.44)_ = 1.70; *p* = 0.09
	Time1	−6.17 (2.55)	*t*_(68.99)_ = −2.42; *p* = 0.02
	Time1^*^ intervention	−14.81 (3.50)	*t*_(68.95)_ = −4.23; *p* < 0.01
	Time2	−5.90 (3.05)	*t*_(72.10)_ = −1.93; *p* = 0.06
	Time2^*^ intervention	−13.85 (4.11)	*t*_(71.73)_ = −3.37; *p* < 0.01
	**Time**	**Condition**	***d***
Effect sizes (*d*)	Time1	PE	0.57
		FR + PE	1.57
	Time2	PE	0.67
		FR + PE	1.39
	**Assessment point**	**Condition**	**M (SD)**
Descriptive statistics	Pre-treatment (t0)	PE	59.49 (9.72)
		FR + PE	64.25 (14.05)
	Post-treatment (t1)	PE	53.91 (14.50)
		FR + PE	42.22 (17.09)
	6-month follow-up (t2)	PE	52.94 (16.94)
		FR + PE	44.65 (19.22)

*PE, Psychoeducation; FR, Functional Relaxation; Time1, Change from pre-treatment (t0) to post-treatment (t1); Time2, Change from pre-treatment (t0) to 6-month follow-up (t2)*.

From t0 to t2, participants receiving PE descriptively reduced their stress-levels, but this effect was statistically insignificant (negative “time2” term; *p* = 0.06). FR + PE was significantly superior to PE from t0 to t2 to reduce stress-levels (negative “time2^*^intervention” term, *p* < 0.05). In PE, the stress-level was reduced on average 5.90 PSQ points at follow-up and in FR + PE, the stress-level was reduced on average 19.75 (5.90 + 13.85) PSQ points.

With regard to the effect sizes (*d*), PE resulted in medium effects both at post-treatment (*d* = 0.57) and at follow-up (*d* = 0.67), whereas FR + PE produced large effects at post-treatment (*d* = 1.57) as well as at follow-up (*d* = 1.39).

### Results for depression and somatization

Tables 3, 4 display the results of the multilevel models for discontinuous change, the effect sizes, and the means as well as the standard deviations for either the PHQ-9 depression (Table 3) scale or the PHQ-15 somatization (Table 4) scale (secondary outcomes) as dependent variable. FR + PE and PE were comparable on these two PHQ scales at t0 (insignificant “intercept^*^intervention” terms; both *p* > 0.05). PE significantly reduced depression from t0 to t1 (negative and significant “time1” term; *p* < 0.05), and the improvement of somatization was very close to significant in the PE condition (negative and insignificant “time1” term; *p* = 0.051). The “time1^*^intervention” terms were negative and attained statistical significance for the PHQ-9 scale and the PHQ-15 scale (both *p* < 0.05). This means that FR + PE was superior to PE at the end of the intervention to reduce depression and somatization. On average, depression was reduced 2.28 PHQ-9 points in PE and 4.45 (2.28 + 2.17) PHQ-9 points in FR + PE from t0 to t1. The average decrease of somatization between t0 and t1was 1.25 PHQ-15 points in PE and 3.30 (1.25 + 2.05) PHQ-15 points in FR + PE.

**Table 3:** Results for the secondary outcome depression measured with the depression scale of the “Patient Health Questionnaire” (PHQ-9).

	**Parameter**	**Estimate (SE)**	**Statistics**
Results of the multilevel model	Intercept	10.03 (0.68)	*t*_(78.54)_ = 14.74; *p* < 0.01
	Intercept^*^ intervention	0.57 (0.95)	*t*_(78.97)_ = 0.60; *p* = 0.55
	Time1	−2.28 (0.72)	*t*_(66.59)_ = −3.16; *p* < 0.01
	Time1^*^ intervention	−2.17 (1.00)	*t*_(67.32)_ = −2.16; *p* = 0.03
	Time2	−1.37 (0.73)	*t*_(72.85)_ = −1.88; *p* = 0.06
	Time2^*^ intervention	−2.61 (1.01)	*t*_(73.38)_ = −2.60; *p* = 0.01
	**Time**	**Condition**	***d***
Effect sizes (*d*)	Time1	PE	0.52
		FR + PE	1.04
	Time2	PE	0.37
		FR + PE	0.95
	**Assessment point**	**Condition**	**M (SD)**
Descriptive statistics	Pre-treatment (t0)	PE	10.03 (4.06)
		FR + PE	10.58 (4.44)
	Post-treatment (t1)	PE	7.91 (3.52)
		FR + PE	5.94 (4.81)
	6-month follow-up (t2)	PE	8.52 (5.05)
		FR + PE	6.38 (5.04)

*PE, Psychoeducation; FR, Functional Relaxation; Time1, Change from pre-treatment (t0) to post-treatment (t1); Time2, Change from pre-treatment (t0) to 6-month follow-up (t2)*.

**Table 4:** Results for the secondary outcome somatization measured with the somatization scale of the “Patient Health Questionnaire” (PHQ-15).

	**Parameter**	**Estimate (SE)**	**Statistics**
Results of the multilevel model	Intercept	10.67 (0.88)	*t*_(59.46)_ = 12.13; *p* < 0.01
	Intercept^*^ intervention	0.32 (1.28)	*t*_(59.47)_ = 0.25; *p* = 0.81
	Time1	−1.25 (0.63)	*t*_(55.33)_ = −2.00; *p* = 0.051
	Time1^*^ intervention	−2.05 (0.89)	*t*_(55.03)_ = −2.29; *p* = 0.03
	Time2	−1.23 (0.73)	*t*_(52.27)_ = −1.69; *p* = 0.10
	Time2^*^ intervention	−2.94 (1.06)	*t*_(53.02)_ = −2.78; *p* = 0.01
	**Time**	**Condition**	***d***
Effect sizes (*d*)	t0−>t1	PE	0.18
		FR + PE	0.73
	t0−>t2	PE	0.19
		FR + PE	0.93
	**Assessment point**	**Condition**	**M (SD)**
Descriptive statistics	Pre-treatment (t0)	PE	10.63 (5.34)
		FR + PE	11.07 (4.59)
	Post-treatment (t1)	PE	9.68 (5.48)
		FR + PE	7.70 (5.20)
	6-month follow-up (t2)	PE	9.62 (6.17)
		FR + PE	6.79 (5.69)

*PE, Psychoeducation; FR, Functional Relaxation; Time1, Change from pre-treatment (t0) to post-treatment (t1); Time2, Change from pre-treatment (t0) to 6-month follow-up (t2)*.

From t0 to t2, participants of the PE condition did not significantly change on any of the two PHQ scales (negative but insignificant “time2” terms; both *p* > 0.05). The negative and significant “time2^*^intervention” terms for the PHQ-9 and PHQ-15 show that FR + PE was more beneficial than PE to reduce depression and somatization from t0 to t2 (both *p* < 0.05). Participants of the PE condition showed an average improvement of 1.37 points on the PHQ-9 depression scale and of 1.23 points on the PHQ-15 somatization scale from t0 to t2, whereas participants randomized to FR + PE had 3.98 (1.37 + 2.61) PHQ-9 points less depression and 4.17 (1.23 + 2.94) PHQ-15 points less somatization at follow-up than at pre-treatment.

Regarding the effect sizes (*d*), participants of PE reached a medium effect size for depression at post-treatment (*d* = 0.52) and a small effect size for depression at follow-up (*d* = 0.37). For depression, FR + PE participants had large effect sizes at post-treatment (*d* = 1.04) as well as at follow-up (*d* = 0.95). Effect sizes for somatization were small in PE at post-treatment (*d* = 0.18) and follow-up (*d* = 0.19). In FR + PE, a medium effect for somatization emerged at post-treatment (*d* = 0.73) and a large effect at follow up (*d* = 0.93).

## Discussion

The current randomized controlled trial compared FR + PE with PE alone. FR + PE was superior to PE in reducing stress-levels at the end of the intervention as well as at 6-month follow-up. Moreover, FR + PE was more beneficial than PE to reduce somatization as well as depression.

The effect sizes for PE to reduce stress-levels as measured with the PSQ (end of intervention: *d* = 0.57; 6-months follow-up: *d* = 0.67) fall into the range of the corresponding effect sizes (end of intervention: *d* = −0.03 to 0.89; follow-up: *d* = −0.10 to 0.78) reported in a meta-analysis on PE for stress-reduction (Van Daele et al., 2012). The effect sizes for PE were, however, higher than the overall effects found in the cited meta-analysis (end of intervention: *d* = 0.27; follow-up: *d* = 0.20). For FR + PE, the effect sizes to reduce stress-levels were large. And large effect sizes have also been shown for mindfulness-based stress-reduction on measures of stress-levels in non-clinical samples (Chiesa and Serretti, 2009; Khoury et al., 2015). Future trials could directly compare FR with mindfulness-based interventions to reveal whether one of these approaches is more beneficial for stress. With regard to depression, FR + PE produced large effects, whereas Khoury et al. (2015) reported that mindfulness-based stress reduction (MBSR) has a moderate effect on measures of depressive symptoms in healthy people. Yet, pre-treatment differences in depressive symptoms might also explain why FR + PE produced large improvements of depression, whereas MBSR resulted in moderate improvements. Our sample had depression scores on the PHQ-9 (Table 3: estimate for FR + PE = 10.60; estimate for PE = 10.03) typical for mild and moderate depression (Kroenke et al., 2001), and the studies on MBSR might have analyzed data from samples with lower depression scores.

A shortcoming of the current study is that no structured or standardized clinical interviews were applied to have information whether and how many of the stressed participants had a depressive or any other mental disorder. Another limitation is the fact that only self-reports were used as outcome measures. Besides subjective stress responses, physiological parameters (e.g., salivary cortisol, heart rate, electrodermal activity) should be considered important complementary data sources to evaluate stress-reduction interventions. Moreover, the use of newer technologies (e.g., smartphones) might be fruitful in future research to measure outcome variables not only at pre-, post-, and follow-up assessments but also as ecological momentary assessments on a daily life basis (e.g., Trull and Ebner-Priemer, 2013; Adams et al., 2017). Moreover, PE might not be the best control condition since psychoeducational conditions “may reduce the likelihood of detecting a true effect in the intervention arms of the trial. Therefore, alternatives to psychoeducational intervention as control groups (for example, attention placebo) are recommended, in order to avoid bias in study outcomes.” (Donker et al., 2009, p. 8).

Despite these limitations the study at hand has also several strengths. The representativeness of the results is increased by the fact that several FR therapists took part in the study. Another strength (with regard to internal validity) is that each therapist treated FR + PE participants as well as PE participants. If the therapists had been different *between* the conditions, this could have negatively affected the internal validity (potential differences in experience, therapeutic skills,…). Nevertheless, it is possible that there were differences between therapists *within* each condition. “That is, even though the therapists are delivering the same specific ingredients, some therapists will do so more skillfully and therefore achieve better outcomes than other therapists delivering the same treatment” (Wampold, 2015, p. 274). A meta-analysis reported that therapist effects amount to *d* = 0.35 in clinical trials (Baldwin and Imel, 2013). Yet, the variance component (for the primary outcome) associated with the therapists was below 1% and statistically insignificant in the current study. A further strength regarding the internal validity is that manuals were used to standardize the delivery of the interventions across the therapists. Although the use of manuals, the same therapists in both conditions, and the randomized design increase the internal validity of the result, the following threats to the internal validity have to be kept in mind, too.

First, the time interval between the pre-treatment assessment and the post-treatment assessment was shorter for PE (approx. 6 weeks) than for FR + PE (10–12 weeks), so that spontaneous remission of elevated stress-levels might have occurred more probably for participants of the FR + PE condition. Yet, this potential confounder is relevant for the short-term treatment effects only, since the time interval from pre-treatment to follow-up (6-months) was the same for both conditions.

Second, the participants of the FR + PE condition received a higher dose (10 × 90 min) than participants of the PE condition (2 × 90 min). Therefore, it is possible that the different dose contributed to the differences between FR + PE and PE in the outcome variables. However, PE shorter in duration has been shown to be more beneficial than PE with longer durations to reduce stress-levels (Van Daele et al., 2012) and, therefore, matching the length might lead to results even more in favor of FR + PE. Moreover, an extended PE of 10 sessions might contain a lot of redundancy and could lead to more drop-outs.

Third, the conditions also differed in supervision: While therapists were supervised for participants of the FR + PE condition, no supervision was provided in the context of the study for participants of the PE condition. Thus, therapist competence and adherence to the treatment manual, for example, might have been higher in FR + PE than in PE. However, inconsistent findings exist whether supervision leads to better outcomes (Watkins, 2011) and a meta-analysis showed that adherence (*r* = 0.02) as well as competence (*r* = 0.07) contribute only marginally to the outcome (Webb et al., 2010).

Future trials with larger samples are needed that take these threats to the internal validity and the other shortcomings into account in order to replicate that FR is beneficial for stress. These further studies should also use structured or standardized clinical interviews to investigate whether FR prevents clinical disorders through stress-reduction.

In summary, the study at hand provides first evidence that the combination of FR and PE is more suited than PE alone for stress. FR might be a potent component of stress interventions. Adding FR to such interventions might better help prevent clinically relevant disorders such as depression or somatization.

## Author contributions

CL was the primary investigator of this study, drafted and revised the manuscript; MG realized the follow-up assessment and revised the manuscript; HS contributed to the statistical analyses and revised the manuscript; AD contributed the study design and revised the manuscript; CP contributed to the study design and revised the manuscript; TP performed the statistical analyses and drafted the manuscript.

### Conflict of interest statement

The authors declare that the research was conducted in the absence of any commercial or financial relationships that could be construed as a potential conflict of interest.
